# Seven COVID-19 Patients Treated with C-Reactive Protein (CRP) Apheresis

**DOI:** 10.3390/jcm11071956

**Published:** 2022-04-01

**Authors:** Fabrizio Esposito, Harald Matthes, Friedemann Schad

**Affiliations:** 1 Intensiv-Notfallmedizin und Kardiologie, Gemeinschaftskrankenhaus Havelhöhe, 14089 Berlin, Germany; 2 Gastroenterologie, Gemeinschaftskrankenhaus Havelhöhe, 14089 Berlin, Germany; harald.matthes@charite.de; 3 Medizinischen Klinik für Gastroenterologie, Infektiologie und Rheumatologie, Charité-Universitätsmedizin, 12203 Berlin, Germany; 4 Institut für Sozialmedizin, Epidemiologie und Gesundheitsökonomie, Charité-Universitätsmedizin, 10117 Berlin, Germany; 5 Interdisziplinäre Onkologie und Supportivmedizin, Gemeinschaftskrankenhaus Havelhöhe, 14089 Berlin, Germany; friedemann.schad@havelhoehe.de

**Keywords:** blood component removal, C-reactive protein, CRP apheresis, COVID-19, multiple organ failure, pulmonary fibrosis, SARS virus

## Abstract

Background: The fulminant course of COVID-19, triggered by severe acute respiratory syndrome coronavirus 2 (SARS-CoV-2), presents with a high mortality rate and still lacks a causative treatment. C-reactive protein (CRP) has been shown to increase dramatically during the disease progression and correlates with deleterious outcomes. Selective CRP apheresis can reduce circulating CRP levels fast and effective. Methods: Seven hospitalized patients with documented severe COVID-19 progression, elevated CRP plasma levels (>100 mg/L) and signs of respiratory failure were treated with CRP apheresis. Two to twelve CRP apheresis sessions were performed generally in 24 h time intervals and depending on CRP plasma levels. Results: All patients had comorbidities. CRP apheresis reduced CRP plasma levels by up to 84% within a few hours, without exhibiting side effects in any patient. Despite signs of severe lung infiltration in all patients, only one patient died. The other patients showed improvements within the chest X-ray after CRP apheresis and were able to recover regardless of intubation and/or ECMO (4 patients). All remaining six patients were discharged from the hospital in good clinical condition. Conclusions: This case series presents a mortality rate of only 14%, which is dramatically lower than expected from the presented CRP levels as well as comorbidities and ventilation requirements. Our clinical observations regarding the here presented seven patients support the hypothesis that CRP is a candidate to be therapeutically targeted in the early stage of severe COVID-19.

## 1. Introduction

C-reactive protein (CRP) is an established biomarker of infection since it was first described in 1930 by Tillet and Francis [1] and can be used as a reliable and fast indicator of the extent of inflammation in the human body. As a classical acute phase protein, CRP rises dramatically within hours of infection or incident and has been shown to activate the complement system via the classical pathway [2] and macrophages via Fcγ-receptors [3,4]. Recently, CRP is not assumed to be only a marker anymore but hypothesized to be an active player in inflammation-induced deleterious tissue processes. This is mainly based on its cytotoxic activity within ischemic and inflamed tissue [5]. After binding to the cell surface, the CRP pentamer may dissociate into monomers suspected to be the pathological agent [6,7,8,9,10]. The question of guilt regarding monomeric or pentameric CRP cannot be clarified by this registry study. Based on the description of the function of the CRP adsorber, we can at least conclude that this medical device adsorbs pentameric CRP [11]. We lack the means to investigate whether it breaks down into the monomer after binding to its target structure.

It has been shown by pathologists [12] that in pulmonary fibrosis, the innate immune system is massively represented, but the adaptive immune system is not. Furthermore, SARS-CoV-2 is hardly detectable. In fact, one would have expected the innate immune system to intervene first and thereafter the adaptive immune system would be activated. With COVID-19, it seems to be the other way around than usual. First, a lot of adaptive immune system can be detected and thereafter the innate immune system causes damage in the lungs.

In the context of the SARS-CoV-2-induced disease COVID-19 it is remarkable that CRP plasma levels rise to an extent similar to bacterial infections [13]. Further, CRP levels correlate with worse prognosis in COVID-19 with an odds ratio of 18.9 [14] and were proven to be a reliable marker for numerous deleterious processes, as, e.g., the need for mechanical ventilation [13,15]. Hence, therapeutically targeting CRP was suggested early on during the pandemic [16,17].

CRP apheresis is an extracorporeal procedure, which decreases CRP plasma levels selectively and with no side effects. Thereby, CRP can finally be targeted therapeutically and specifically [18,19,20,21]. It was recently introduced as a potential treatment of severe SARS-CoV-2-induced pneumonia [16,22]. After three case reports describing individual healing attempts [22,23,24] the “C-reactive protein Apheresis in COVID” (CACOV; DRKS00024376) registry was initiated, which already led to the publication of a case series by another participating center [25]. The seven severe COVID-19 patients treated there survived in good health. Further and based on the results of the CACOV registry, the randomized “C-reactive protein Apheresis for Attenuation of Pulmonary, Myocardial and/or Kidney Injury in COVID-19” (CAPMYKCO; NCT04898062) trial was designed.

From the experience with CRP apheresis in myocardial infarction, we concluded that the earliest possible time for the use of CRP apheresis should be aimed for, which we assume to be in the first 72 h after the onset of severe COVID-19. A publication by Mueller et al. reported that a CRP increase after hospitalization of 13 mg/L within 48 h indicates a poor prognosis including invasive ventilation [13]. The same was shown for CRP levels on admission to the hospital. Here, the threshold value is approximately 146 mg/L. Another publication puts the CRP cutoff value at around 97 mg/L [26]. This report summarizes the treatment of seven COVID-19 patients suffering from severe SARS-CoV-2-induced pneumonia treated by CRP apheresis.

## 2. Materials and Methods

### 2.1. CACOV Registry

The CACOV registry is a post market clinical follow up to investigate the reduction of C-reactive protein (CRP) by selective C-reactive protein apheresis in patients with COVID-19 and highly elevated CRP plasma levels. This analysis includes the first seven patients with severe SARS-CoV-2-induced pneumonia and signs of respiratory failure who exceeded CRP plasma levels of 100 mg/L and who could be subjected to CRP apheresis in the early phase (first 72 h) of severe COVID-19. Patients were treated between March and May 2021. All patients provided written informed consent. In this registry, the only inclusion criterion is, that the patient with positive PCR-test for SARS-CoV-2 should have elevated CRP and be treated with CRP apheresis. All patients required intensive care.

### 2.2. CRP Apheresis

A regenerative single adsorbent system was used for CRP apheresis (PentraSorb^®^ CRP; Pentracor GmbH, Hennigsdorf, Germany). Apheresis is performed in cycles, alternating between loading the adsorber with patient plasma and regeneration, which follows a fixed sequence of wash solutions (≥100 mL NaCl, ≥60 mL glycine/HCl, ≥80 mL PBS and ≥80 mL NaCl). The flow of plasma and wash solutions during apheresis was controlled by an automated plasma flow management software module (ADAsorb, medicap clinic GmbH, Ulrichstein, Germany). Blood collection was performed via central venous access because of the high clotting tendency of COVID-19 patients. Plasma separation was performed with a centrifuge (SpectraOptia, TerumoBCT, Denver, CO, USA). For plasma separation, blood was anticoagulated with 1:15 citrate buffer (Anticoagulant Citrate Dextrose Solution A = ACD-A). Plasma flow through the adsorber was 25 to 40 mL/min. Blood flow ranged from 47 to 90 mL/min. Up to 8000 mL of plasma was processed during the treatments, preferably in cycles (change of loading and regeneration of the adsorber) of 1000 and 500 mL, respectively. For routine monitoring of apheresis, blood was drawn from the extracorporeal circulation before and after each treatment to determine the CRP concentration.

### 2.3. Ventilation Scheme

In hypoxic patients or SpO2 < 90%, we started oxygen therapy by goggle or mask, high-flow oxygen therapy and later noninvasive ventilation if necessary, taking into account that delay of intubation in the absence of response to non-invasive ventilation (NIV) worsens the prognosis. In parallel, we performed restrictive fluid therapy in circulatory stable patients.

In invasively ventilated patients, we aimed for a protective ventilation strategy with Vt ≤ 6 mL/kg, driving pressure < 15 cm H_2_O, end-inspiratory airway pressure (pInsp) < 28 cm H_2_O and the PEEP setting was based on the so-called high-PEEP table. We performed early abdominal positioning with at least 16 h abdominal positioning intervals. In case of refractory hypoxemia, inhalative application of nitric oxide (NO) was performed and recruitment maneuvers were considered, if necessary, after sonography/CT/EIT. In case of persistent hypoxia, after exhaustion of further therapeutic measures, exclusion of contraindications and consultation with relatives regarding the patient’s wishes, the use of venovenous extracorporeal membrane oxygenation (ECMO) was performed.

### 2.4. Contraindications

Liver failure, hepatic insufficiency, as citrate is used as an anticoagulant in the centrifuge for plasma separation. If citrate is not metabolized quickly by the liver, the blood becomes acidic. Therefore, the liver function should be sound.

## 3. Results

### 3.1. Patient Characteristics

We used defined criteria for the selection of patients for this case series. Patients had to be diagnosed with the severe course of COVID-19 and the first CRP apheresis had to be performed a maximum 72 h after the onset of this severe course. The severe disease course was defined by the requirement for oxygen supply, a CRP plasma concentration > 100 mg/L, a poor overall condition and visible COVID-19 infiltrates in the chest X-ray/CT.

Patient characteristics are summarized in Table 1. Age, sex, preexisting and concurrent diseases, length of hospital stay (7–75 days), ventilation therapy (exact type indicated) as well as treatment and apheresis data are shown. All seven patients required either non-invasive oxygen supply or invasive ventilation therapy and suffered from concomitant diseases. Six of seven patients suffered from bacterial superinfection during the hospital stay and therefore received antibiotic therapy.

**Figure 1:**
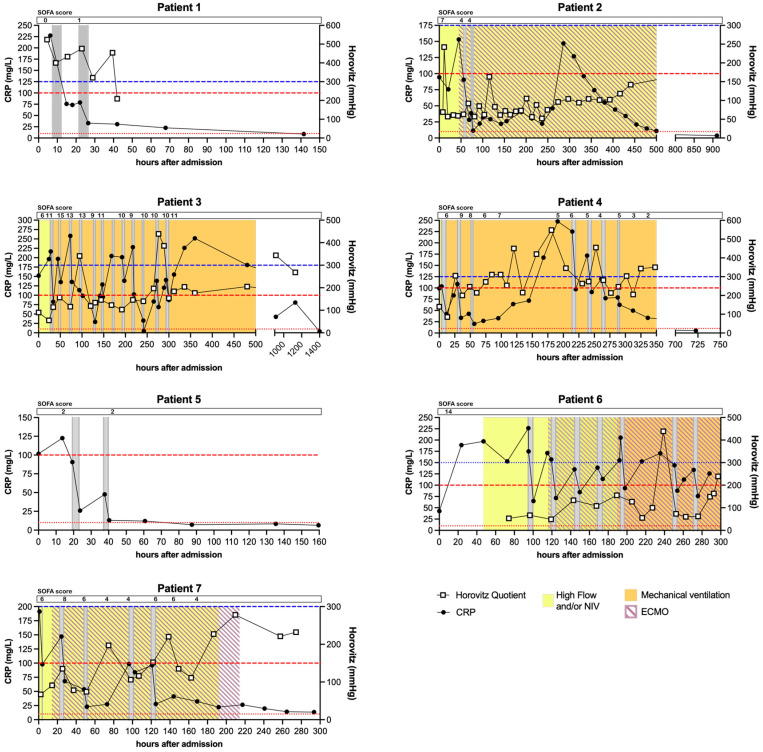
**CRP kinetics, Horovitz Quotient and SOFA scores of all patients of the case series.** CRP was measured at least every 24 h in all patients during in-hospital stay. Red lines indicate 10 mg/L and 100 mg/L as reference for normal levels and cut-off for severe COVID-19 progression respectively. Blue line indicates 300 mmHg as cut-off for an acute lung injury measured by the Horovitz Quotient. Grey bars indicate apheresis treatments. Ventilation by High Flow or Non-Invasive Ventilation (NIV) is marked in yellow. Mechanical ventilation is marked in orange and extracorporeal membrane oxygenation (ECMO) is marked with purple stripes. No ventilation and nasal cannula are not indicated (for details see Table 1). SOFA scores are displayed at corresponding timepoints. For patient 6 the SOFA score was only determined once.

Patients received 2–12 apheresis sessions depending on their CRP concentration and overall condition. CRP depletion rates ranged from 18–84% per session, strongly depending on the processed plasma volume, stage of CRP synthesis (acute phase) and initial concentration (see Figure 1 for detailed kinetics).

Only one patient (6) died, all others could be discharged in good clinical condition without the requirement of further ventilation.

### 3.2. CRP Kinetics

Figure 1 depicts the CRP plasma kinetics of each patient. CRP levels were elevated (>100 mg/L) on admission in all patients except patients 2 and 6, who then showed rising levels within the first 20–50 h after admission. CRP apheresis sessions (grey bars) always led to a pronounced decrease in CRP levels. Patients 1, 4, 5 and 7 were treated with apheresis until CRP declined below 100 mg/L and stayed low. Patients 2 and 3 showed a marked increase in CRP levels after their last apheresis session (~300–350 h after admission), which can be correlated with the diagnosed bacterial superinfection (Procalcitonin levels of other patients in Supplementary Figures S1–S6) and was not treated with CRP apheresis but with antibiotics. CRP levels declined before release. CRP levels re-bound steadily after each CRP apheresis session in patient 6 and could not be maintained below 100 mg/L until the patient unfortunately died.

### 3.3. X-ray/CT Chest Scans

All patients received an initial chest X-ray/CT upon hospital admission and subsequent follow-up scans during or after their stay as follow up, depending on ventilation interventions and overall condition. The time between scans varied from 5 to 115 days.

**Figure 2:**
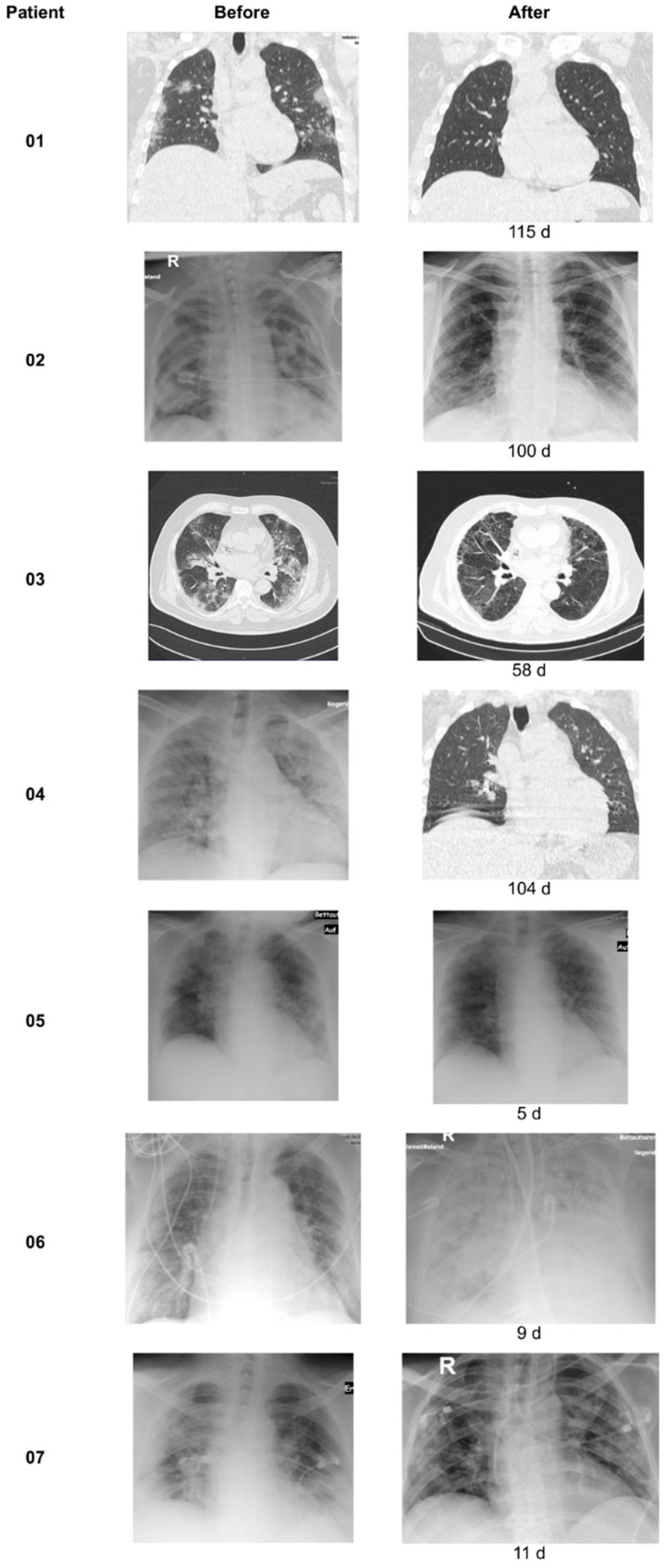
**X-ray/CT chest scans.** Chest scans were performed before and after treatment/as follow up. The time (days) between the different scans is indicated at the second chest scan.

All surviving patients showed improvements within the follow-up X-ray scans after apheresis sessions (Figure 2). Even patient 6 initially showed lung infiltration improvement after the 5th apheresis session, before deteriorating to multi-organ failure and death.

### 3.4. Respiratory and Laboratory Parameters

**Figure 3:**
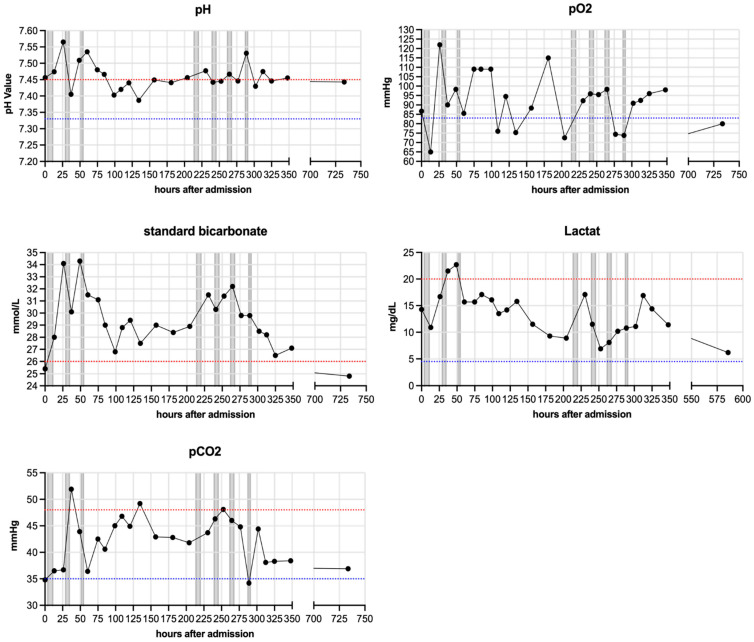
**Respiratory parameters of one patient (patient 4).** pH, standard HCO_3_^−^, lactate, arterial pCO_2_ and pO_2_ were measured regularly for each patient and are depicted here representatively for patient 4. Blue lines indicate minimum baseline and red lines maximum baseline for each normal range. Grey bars indicate apheresis treatments.

**Figure 4:**
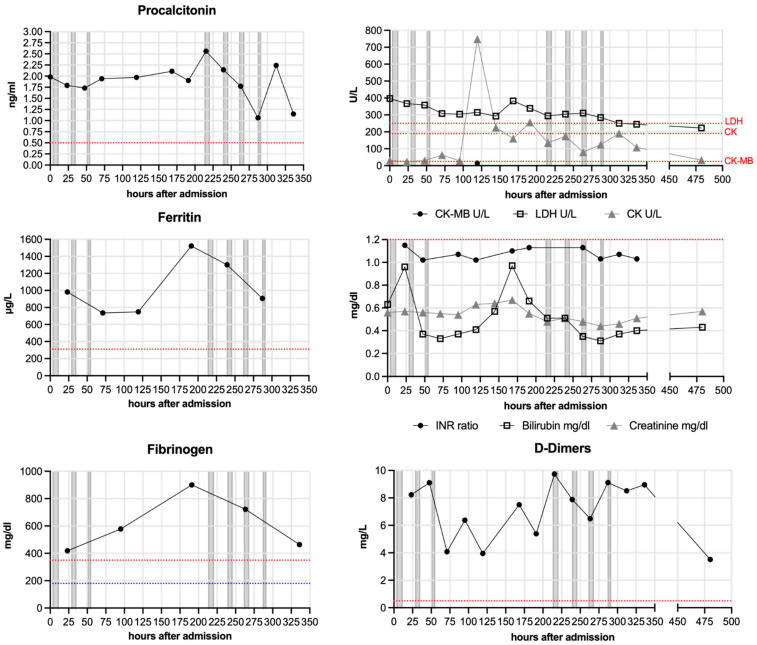
**Other laboratory parameters of one patient (patient 4).** Procalcitonin, CK-MB, CK, LDH, Ferritin, INR ratio, Bilirubin, Creatinine, Fibrinogen and D-Dimers were measured regularly for each patient and are depicted here representatively for patient 4. Blue lines indicate minimum baseline and red lines maximum baseline for each normal range. Grey bars indicate apheresis treatments.

Figure 3 summarizes respiratory parameters in one representative patient (patient 4), Figure 4 summarizes other laboratory results of the same patient.

All patients except patient 6 markedly improved their respiratory parameters during hospitalization (Figure 3). Patient 4 specifically showed metabolic alkalosis (elevated pH and elevated HCO_3_^−^). He was mechanically ventilated for 24 days (~150–570 h after admission) and showed elevated lactate levels, which normalized over time.

The other laboratory values (Figure 4) included Procalcitonin, which was elevated in all patients except patient 5, who solely did not receive antibiotic treatment. Four patients had a laboratory constellation of infections at or shortly after admission. Therefore, they were not treated by immunomodulation and immunosuppression. Other parameters (Ferritin, Fibrinogen, D-Dimers, LDH and CK) were also elevated in numerous patients (including 4), but decreased during the hospital stay and were normal upon discharge. Only patient 6 developed signs of multiple organ failure showing rising levels of Bilirubin, Creatinine, LDH and Procalcitonin as well as a severe acidosis. Patient 6 had a SOFA score of 14 at admission to our ICU.

### 3.5. Control Cohort

At the start of the CACOV registry, we had treated 30 severe COVID-19 patients according to the valid guidelines. Of this cohort, three developed CRP < 100 mg/L. All of them survived. The other 27 developed CRP > 100 mg/L. Thirteen of them died which is 48%.

## 4. Discussion

The severe course of COVID-19, with its high mortality rate, is fundamentally caused by an excessive immune response, often-called cytokine storm, which mediates the destruction of mainly pulmonary tissue [27,28]. CRP has been established as one of the key effector molecules of this process [5,15,29]. A recent review outlines that and how CRP provides for the disposal of hypoxic cells [5]. This finding is supported by the CRP apheresis after AMI-1 (CAMI-1) clinical trial [18,19,20,21]. The CAMI-1 trial clearly and significantly demonstrates, based on results in the control group, that the more CRP the patient synthesizes, the greater the damage to the heart (infarct size, ejection fraction, wall motility). It has been described that this is a dose-dependent effect. Therefore, we suspected that reducing its circulating levels is the next logical step in order to inhibit the deleterious destruction of lung cells, which could recover during ventilation and with more time. CRP apheresis presents the first therapeutic opportunity to target CRP selectively and quickly.

Three COVID-19 patients have been treated with CRP apheresis as individual healing attempts before [22,23,24]. Subsequently, the CACOV registry started and is running at several centers throughout Germany. Here, we publish a case series of seven patients, with the following characteristics.

All patients reported here were diagnosed with the severe course of COVID-19 and CRP apheresis was initiated within 72 h of this onset. Further, CRP serum levels exceeded 100 mg/L. CRP apheresis significantly decreased the elevated CRP plasma levels in all seven patients, which supports the efficacy of this therapy and is in line with all patients treated with CRP apheresis so far [19,20,21,22,23,24]. All patients exhibited signs of respiratory or metabolic alkalosis, which is known to occur in COVID-19 [30].

Apart from one patient (6), who unfortunately died of organ failure 14 days after hospital admission, all patients recovered from COVID-19 and could be discharged in good clinical condition after 2–12 CRP apheresis sessions.

All patients besides patient 5 showed elevated Procalcitonin levels and had to be treated with antibiotics. Further, three patients received Dexamethasone/Colchicine upon hospital admission (Table 1), which is known to lower CRP plasma levels to a certain extent for a short period. However, this was not robustly visible within the individual CRP kinetics (Figure 1) and the performed CRP apheresis, with an up to 84% reduction within hours, was definitely more efficient in decreasing CRP plasma levels.

Based on the existing literature and our results, we assume that fluid retention in the lungs and the hypoxia induced by it provide for the induction of CRP [5,23]. The CRP kinetics then depends on the kinetics of the fluid retention and also on the kinetics of the disappearance of the fluid.

Focusing on the CRP kinetics, all patients depicted maximum CRP levels over 120 mg/L. The significant correlation of the increase in CRP as well as the maximum CRP amount with mortality has been widely established so far [14,31,32,33]. In detail, in one study a 108 mg/L CRP cut-off led to a higher mortality of 32% vs. 18% [15]. In another report, a maximum CRP serum concentration of >100 mg/L was associated with either progressive or severe COVID-19 with a mortality of up to 59% vs. 4% in the mild group (<100 mg/L) [13]. In our in-house control cohort, 48% of severe COVID-19 patients which developed CRP > 100 mg/L died. However, in our case series, the mortality rate was only 14% and thereby dramatically lower than expected from the presented CRP levels as well as co-morbidities and ventilation requirements. All of them are in good health in February 2022 and, therefore, at least 9 months after discharge from the hospital. This is remarkable, as reports from Germany [34] and the US [35] showed that there is a significantly increased risk for mortality over the next 6 or 12 months, respectively. Our clinical observations regarding the here presented seven patients support the hypothesis that CRP should be therapeutically targeted in COVID-19.

### 4.1. Limitations

In order to conclusively prove that CRP apheresis is the therapy of choice in severe COVID-19 courses, randomized controlled trials are urgently needed.

### 4.2. Conclusions

Our data support the hypothesis that the damage to the lung caused in severe COVID-19 appears to result primarily from excessive CRP-mediated disposal of oxygen-depleted lung areas. CRP apheresis starting early after patient admission, may potentially be an effective treatment COVID-19 and save lung tissue. Additional registry data and randomized controlled clinical trials are required.

## Figures and Tables

**Table 1:** **Patient characteristics.** The table shows age, sex, concomitant diseases, respiratory supply, in-hospital length of stay and treatment parameters of all 7 patients. All patients had documented SARS-CoV-2-induced pneumonia and showed signs of respiratory failure. Patient 6 had acute renal failure (AKI) shortly before his demise. F female, M male, HF High Flow, NIV non-invasive ventilation, M ventilation mechanical ventilation, y yes, n no, ECMO extracorporeal membrane oxygenation, Dex Dexamethasone, Col Colchicine, ABs antibiotics, HVL Gemeinschaftskrankenhaus Havelhöhe (our hospital).

Patient Number	Age	Sex	Hospitalized (Days)	Type of Ventilation (Days)	Treatment	CRP Apheresis (*n*)	CRP Depletion	Processed Plasma Volume	X-ray Improvement after Apheresis	Survival	Preexisting Diseases	Adipositas	Diabetes (Type)	Cardiovascular	Other	Concurrent Diseases
1	33	F	7	Nasal cannula (3)	Dex, Col, ABs	2	58–67%	7.5 L	y	y		y	n	y	Factor V Leiden, Factor II Mutation, microcytic anemia	Bacterial s.infection
2	54	F	44	HF and NIV (24) ECMO (19)	ABs	2	69–70%	7.5–8 L	y (1st)	y		y	Type 2	y		Bacterial s.infection
3	69	M	75	HF (3) M ventilation (72)	ABs	12	13–84%	6.5–10 L	y (6th)	y		y	Type 2	y		Bacterial s.infection
4	47	M	39 (34 HVL)	HF (1) M ventilation (24)	ABs	7	20–69%	5–10 L	y (2nd)	y		y	n	y		Bacterial s.infection
5	72	F	9	Nasal cannula (8)	Dex, Col	2	71–72%	4–6 L	y (2nd)	y		y	n	n	Alcohol abusement	
6	77	M	13	HF and NIV (7) M Ventilation (5) ECMO (8)	ABs	7	18–71%	7–9 L	y (5th) Then worse until death	n		n	Type 2	y	multimorbid	Bacterial s.infection, AKI
7	53	F	17	Nasal cannula (7) HF (10) ECMO (10)	Dex, Col, ABs	5	15–71%	6–8.5 L	y (5th)	y		n	n	y		Viral hepatitis, Bacterial s.infection
Mean	57.9		29.1			5.3										

## Data Availability

The original contributions presented in the study are included in the article/Supplementary Materials, further inquiries can be directed to the corresponding author.

## Supplementary Materials

The following supporting information can be downloaded at: https://www.mdpi.com/article/10.3390/jcm11071956/s1, Figures S1–S6: Respiratory and laboratory parameters of Patient 1–3 and 5–7.
