# Supplementary material for: Seven COVID-19 Patients Treated with C-Reactive Protein (CRP) Apheresis
Source: J Clin Med. 2022 Apr 1;11(7):1956. doi: 10.3390/jcm11071956 (PMC8999883; doi:10.3390/jcm11071956)
Supplement: Supplementary file 1 [file jcm-11-01956-s001.zip › jcm-1635570-supplementary.pdf]

Supplementary Material

1 Supplementary Figures

Patient 1

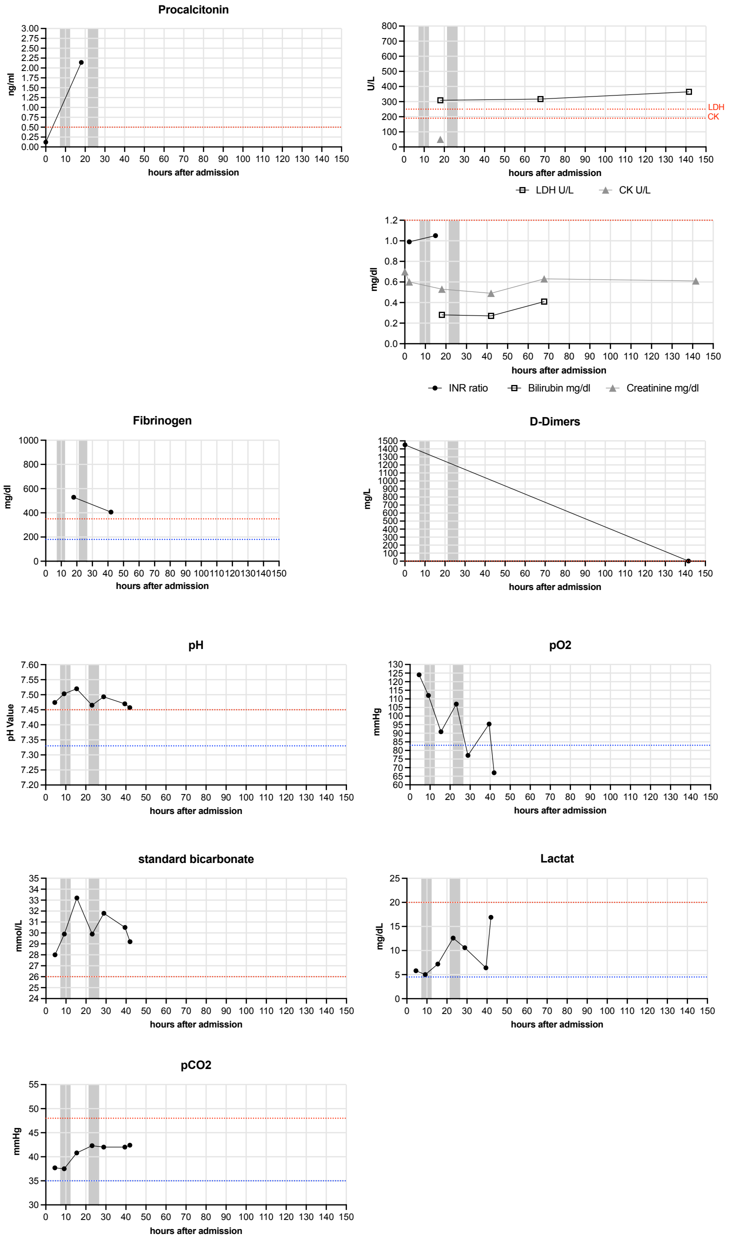

Supplementary Figure S1: Respiratory and laboratory parameters of Patient 1. Procalcitonin, CK, LDH, INR ratio, Bilirubin, Creatinine, Fibrinogen, D-Dimers pH, standard $HCO3^-$, lactate, arterial pCO_2 and pO_2 were measured regularly for each patient and are depicted here. Blue lines indicate minimum baseline and red lines maximum baseline for each normal range. Grey bars indicate apheresis treatments.

Patient 2

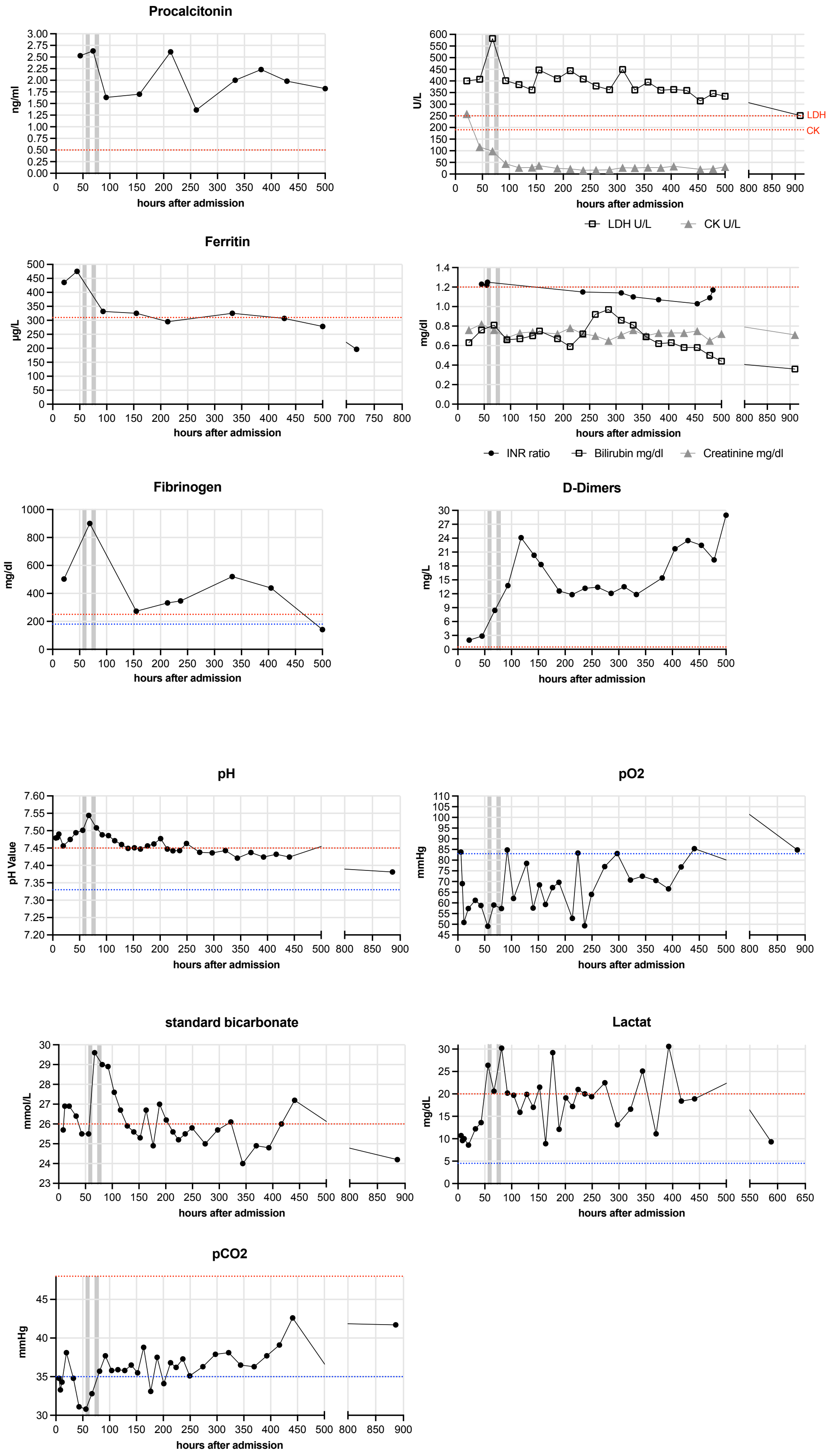
Procalcitonin
ng/ml
hours after admission
U/L
LDH
CK
hours after admission
LDH U/L
CK U/L
Ferritin
µg/L
hours after admission
mg/dl
hours after admission
INR ratio
Bilirubin mg/dl
Creatinine mg/dl
Fibrinogen
mg/dl
hours after admission
D-Dimers
mg/L
hours after admission
pH
pH Value
hours after admission
pO2
mmHg
hours after admission
standard bicarbonate
mmol/L
hours after admission
Lactat
mg/dL
hours after admission
pCO2
mmHg
hours after admission

Supplementary Figure S2: Respiratory and laboratory parameters of Patient 2. Procalcitonin, CK-MB, CK, LDH, Ferritin, INR ratio, Bilirubin, Creatinine, Fibrinogen, D-Dimers pH, standard $HCO3^-$, lactate, arterial pCO_2 and pO_2 were measured regularly for each patient and are depicted here. Blue lines indicate minimum baseline and red lines maximum baseline for each normal range. Grey bars indicate apheresis treatments.

Patient 3

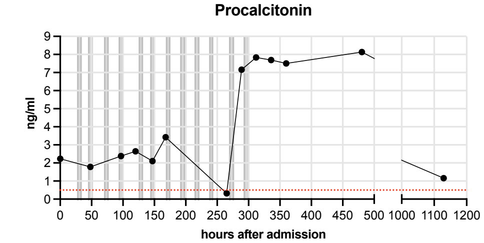
Procalcitonin
ng/ml
hours after admission

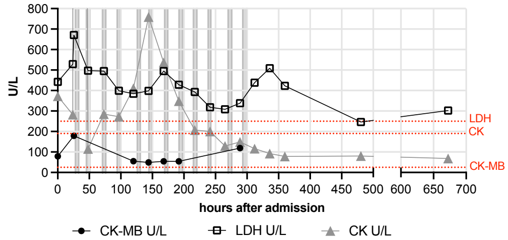
U/L
LDH
CK
CK-MB
hours after admission
CK-MB U/L
LDH U/L
CK U/L

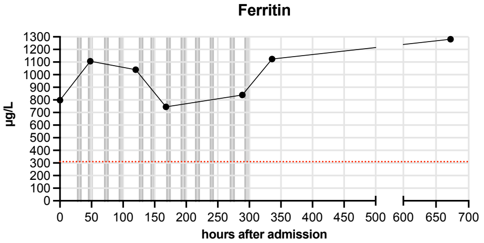
Ferritin
µg/L
hours after admission

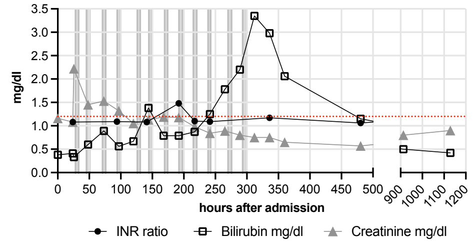
mg/dl
hours after admission
INR ratio
Bilirubin mg/dl
Creatinine mg/dl

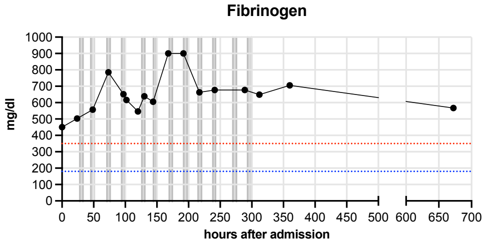
Fibrinogen
mg/dl
hours after admission

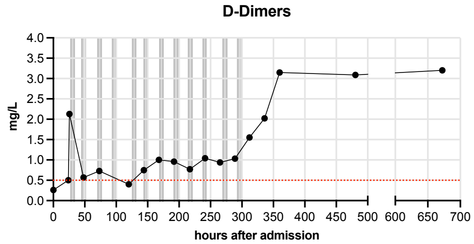
D-Dimers
mg/L
hours after admission

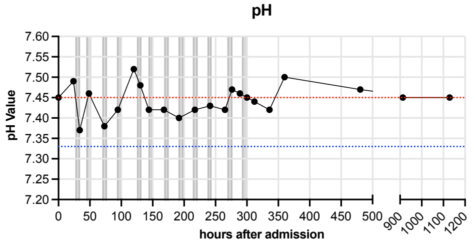
pH
pH Value
hours after admission

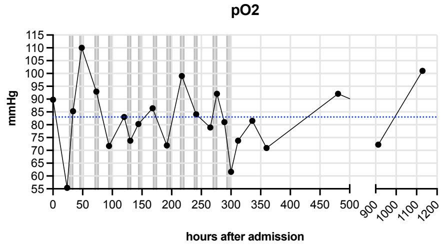
pO2
mmHg
hours after admission

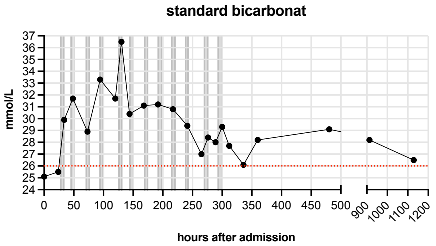
standard bicarbonat
mmol/L
hours after admission

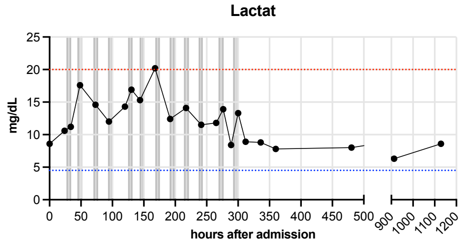
Lactat
mg/dL
hours after admission

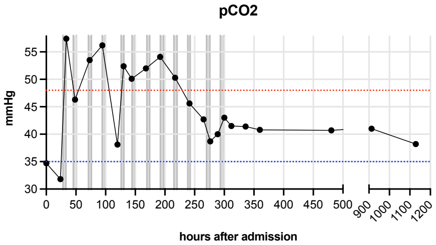
pCO2
mmHg
hours after admission

Supplementary Figure S3: Respiratory and laboratory parameters of Patient 3. Procalcitonin, CK-MB, CK, LDH, Ferritin, INR ratio, Bilirubin, Creatinine, Fibrinogen, D-Dimers pH, , standard $HCO3^-$, lactate, arterial pCO_2 and pO_2 were measured regularly for each patient and are depicted here. Blue lines indicate minimum baseline and red lines maximum baseline for each normal range. Grey bars indicate apheresis treatments.

Patient 5

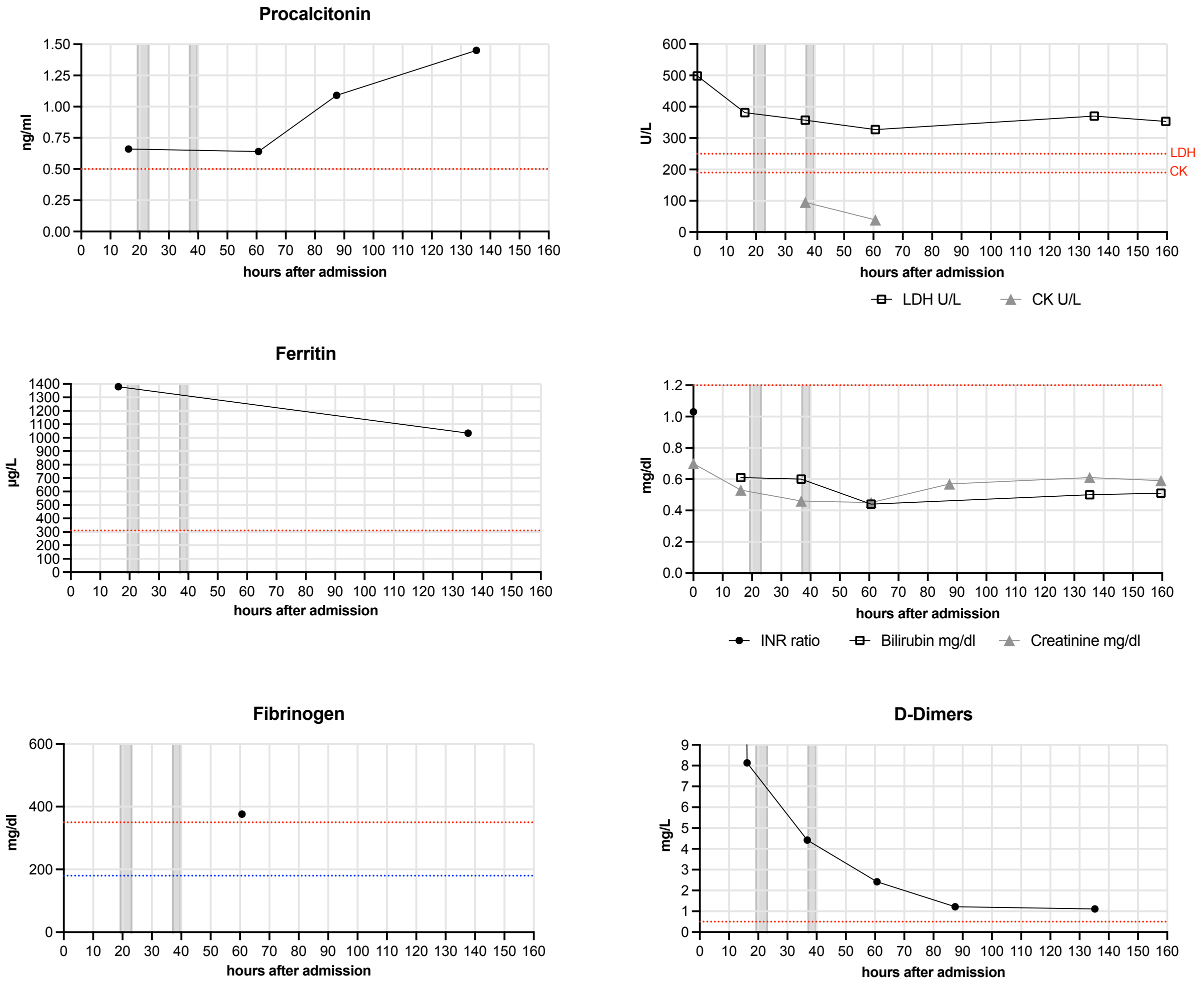

Supplementary Figure S4: Respiratory and laboratory parameters of Patient 5. Procalcitonin, CK, LDH, Ferritin, INR ratio, Bilirubin, Creatinine, Fibrinogen, D-Dimers were measured regularly for each patient and are depicted here. Blue lines indicate minimum baseline and red lines maximum baseline for each normal range. Grey bars indicate apheresis treatments.

Patient 6

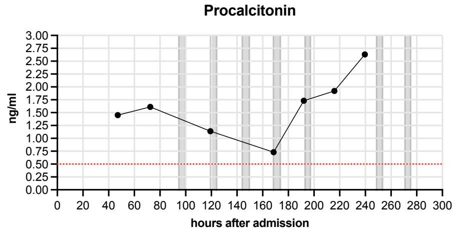
Procalcitonin
ng/ml
hours after admission

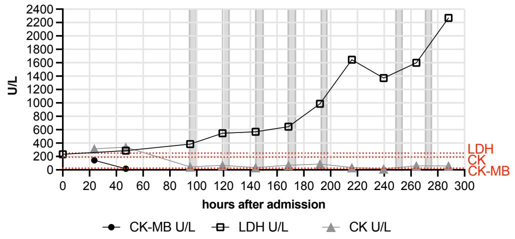
U/L
LDH
CK
CK-MB
hours after admission
CK-MB U/L
LDH U/L
CK U/L

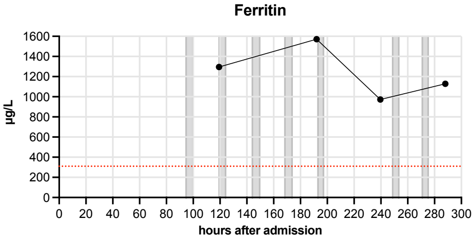
Ferritin
µg/L
hours after admission

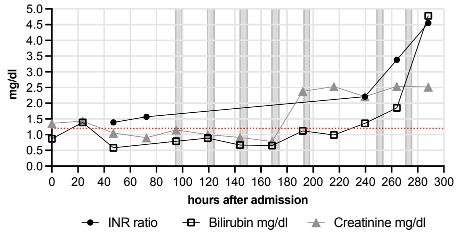
mg/dl
hours after admission
INR ratio
Bilirubin mg/dl
Creatinine mg/dl

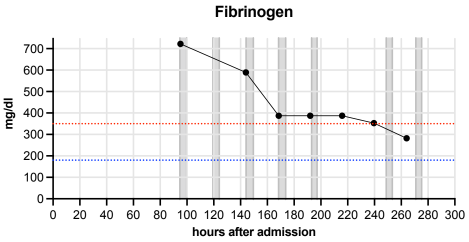
Fibrinogen
mg/dl
hours after admission

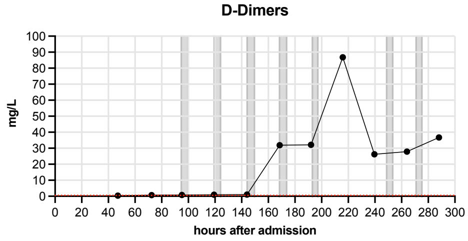
D-Dimers
mg/L
hours after admission

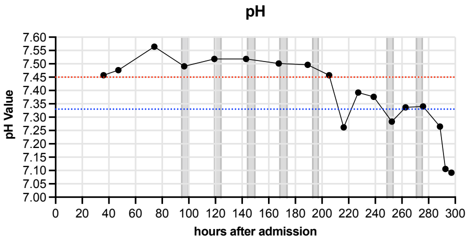
pH
pH Value
hours after admission

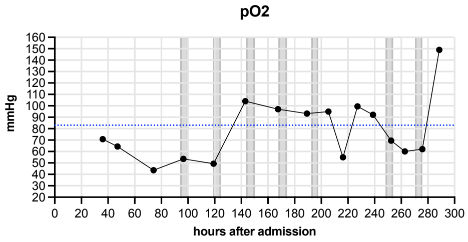
pO2
mmHg
hours after admission

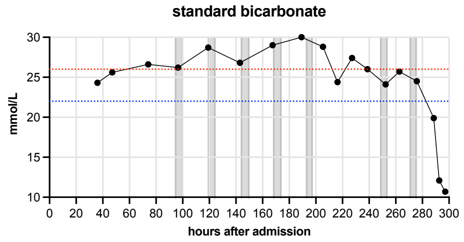
standard bicarbonate
mmol/L
hours after admission

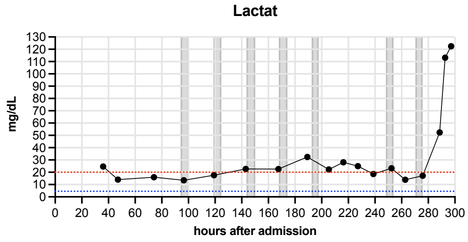
Lactat
mg/dL
hours after admission

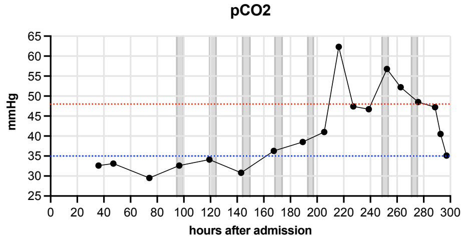
pCO2
mmHg
hours after admission

Supplementary Figure S5: Respiratory and laboratory parameters of Patient 6. Procalcitonin, CK-MB, CK, LDH, Ferritin, INR ratio, Bilirubin, Creatinine, Fibrinogen, D-Dimers pH, standard $HCO3^-$, lactate, arterial pCO_2 and pO_2 were measured regularly for each patient and are depicted here. Blue lines indicate minimum baseline and red lines maximum baseline for each normal range. Grey bars indicate apheresis treatments.

Patient 7

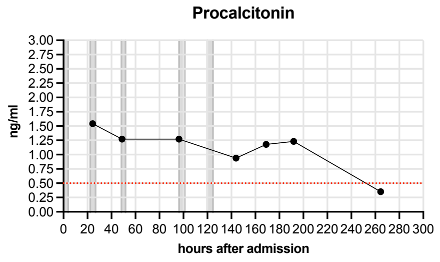
Procalcitonin
ng/ml
hours after admission

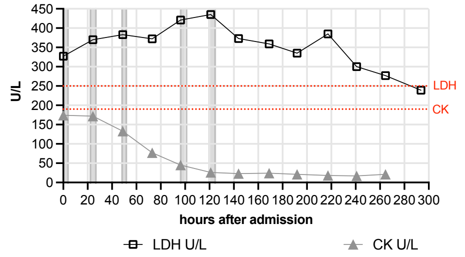
U/L
LDH
CK
hours after admission
LDH U/L
CK U/L

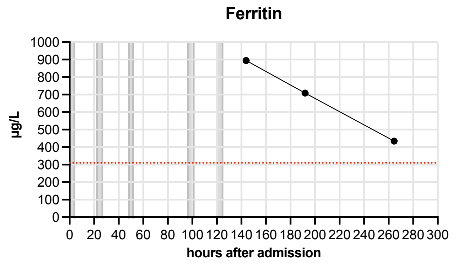
Ferritin
µg/L
hours after admission

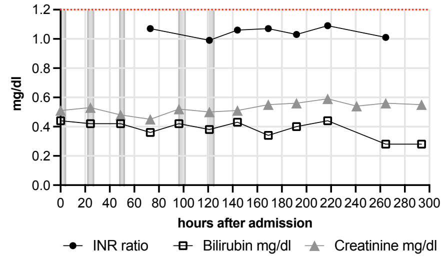
mg/dl
hours after admission
INR ratio
Bilirubin mg/dl
Creatinine mg/dl

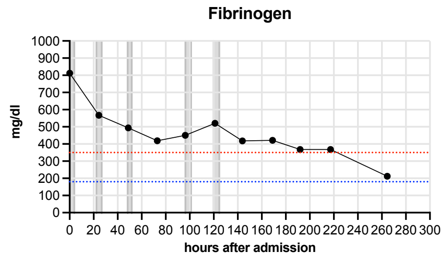
Fibrinogen
mg/dl
hours after admission

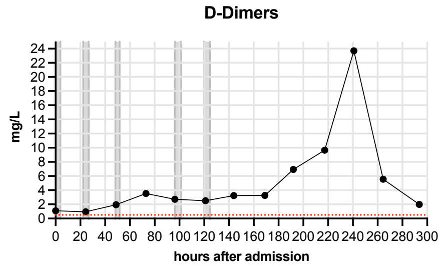
D-Dimers
mg/L
hours after admission

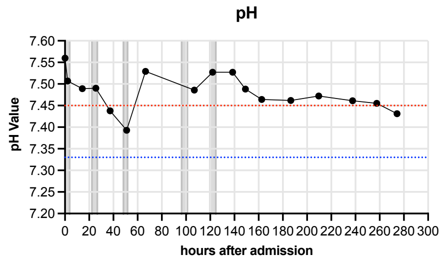
pH
pH Value
hours after admission

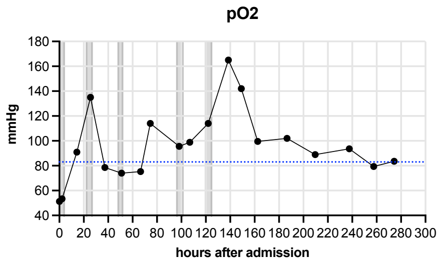
pO2
mmHg
hours after admission

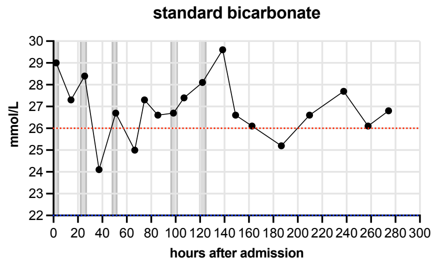
standard bicarbonate
mmol/L
hours after admission

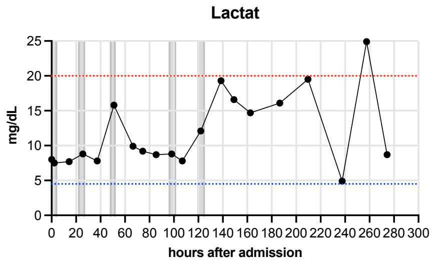
Lactat
mg/dL
hours after admission

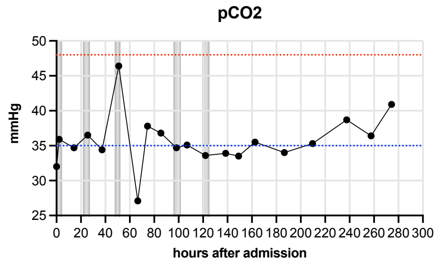
pCO2
mmHg
hours after admission

Supplementary Figure S6: Respiratory and laboratory parameters of Patient 7. Procalcitonin, CK-MB, CK, LDH, Ferritin, INR ratio, Bilirubin, Creatinine, Fibrinogen, D-Dimers pH, standard $HCO3^-$, lactate, arterial pCO_2 and pO_2 were measured regularly for each patient and are depicted here. Blue lines indicate minimum baseline and red lines maximum baseline for each normal range. Grey bars indicate apheresis treatments.
